# Selective Activation of ZAK β Expression by 3-Hydroxy-2-Phenylchromone Inhibits Human Osteosarcoma Cells and Triggers Apoptosis via JNK Activation

**DOI:** 10.3390/ijms21093366

**Published:** 2020-05-09

**Authors:** Chien-Yao Fu, Ing-Shiow Lay, Marthandam Asokan Shibu, Yan-Shen Tseng, Wei-Wen Kuo, Jaw-Ji Yang, Tso-Fu Wang, B. Mahalakshmi, Yu-Lan Yeh, Chih-Yang Huang

**Affiliations:** 1 Orthopaedic Department, Armed Forces General Hospital, Taichung 411, Taiwan; chien-yao@803.org.tw; 2 Department of Orthopaedic, National Defense Medical Center, Taipei 114, Taiwan; 3 School of Post-Baccalaureate Chinese Medicine, China Medical University, Taichung 404, Taiwan; d1540@mail.bh.cmu.edu.tw; 4 Chinese Medicine Department, China Medical University Beigang Hospital, Yunlin 651, Taiwan; 5 Cardiovascular and Mitochondrial Related Disease Research Center, Hualien Tzu Chi Hospital, Buddhist Tzu Chi Medical Foundation, Hualien 97004, Taiwan; shibu@tzuchi.com.tw; 6 Graduate Institute of Biomedical Science, China Medical University, Taichung 404, Taiwan; jly63203@gmail.com; 7 Department of Biological Science and Technology, China Medical University, Taichung 404, Taiwan; wwkuo@mail.cmu.edu.tw; 8 School of Dentistry, Chung-Shan Medical University, Taichung 402, Taiwan; jjyang@csmu.edu.tw; 9 Department of Hematology and Oncology, Hualien Tzu Chi Hospital, Buddhist Tzu Chi Medical Foundation, Hualien 97004, Taiwan; tfwang@tzuchi.com.tw; 10 School of Medicine Tzu Chi University, 701, Section 3, Chung-Yang Road, Hualien 97004, Taiwan; 11 Institute of Research and Development, Duy Tan University, Da Nang 550000, Vietnam; b.mahalakshmibharath@duytan.edu.vn; 12 Department of Pathology, Changhua Christian Hospital, Changhua 500, Taiwan; 13 Department of Medical Technology, Jen-Teh Junior College of Medicine, Nursing and Management, Miaoli 356, Taiwan; 14 Department of Medical Research, China Medical University Hospital, China Medical University, Taichung 404, Taiwan; 15 Department of Biotechnology, Asia University, Taichung 41354, Taiwan; 16 Center of General Education, Buddhist Tzu Chi Medical Foundation, Tzu Chi University of Science and Technology, Hualien 97004, Taiwan

**Keywords:** osteosarcoma, 3-Hydroxy-2-phenylchromone, ZAKβ, apoptosis

## Abstract

Although various advancements in radical surgery and neoadjuvant chemotherapy have been developed in treating osteosarcoma (OS), their clinical prognosis remains poor. A synthetic chemical compound, 3-hydroxylflavone, that is reported to regulate ROS production is known to inhibit human bone osteosarcoma cells. However, its role and mechanism in human OS cells remains unclear. In this study, we have determined the potential of 3-Hydroxy-2-phenylchromone (3-HF) against OS using human osteosarcoma (HOS) cells. Our previous studies showed that Zipper sterile-alpha-motif kinase (ZAK), a kinase member of the MAP3K family, was involved in various cellular events such as cell proliferation and cell apoptosis, and encoded two transcriptional variants, ZAKα and β. In this study, we show that 3-HF induces the expression of ZAK and thereby enhances cellular apoptosis. Using gain of function and loss of function studies, we have demonstrated that ZAK activation by 3-HF in OS cells is confined to a ZAKβ form that presumably plays a leading role in triggering ZAKα expression, resulting in an aggravated cancer apoptosis. Our results also validate ZAKβ as the predominant form of ZAK to drive the anticancer mechanism in HOS cells.

## 1. Introduction

Osteosarcoma (OS) is a rare cancerous tumor that commonly affects children and young adults [1]. Boys are twice as likely to have osteosarcoma as girls, and most cases of osteosarcoma involve the bones around the knee [2]. Osteosarcoma (OS) is also the most common histological form of primary bone cancer and sometimes spreads elsewhere [3]. In spite of advancements in the therapeutic strategies such as radical surgery and neoadjuvant chemotherapy, the clinical outcome of OS remains poor [4]; therefore, therapies that are more efficient must be explored. Our previous reports show that the chemotherapeutic agent doxorubicin induces cell apoptosis and attenuates cell viability of human bone OS cells by triggering Zipper sterile-alpha-motif kinase (ZAK) expression [5]. However, doxorubicin, being a chemotherapeutic agent, causes serious side effects, and therefore finding an alternative that induces ZAK expression is desirable in treating human OS.

A synthetic flavonol 3-Hydroxy-2-phenylchromone (3-HF) [6] has been shown to inhibit endogenous Aurora B and hinder cancer cell growth [7]. In human bone osteosarcoma cells, U2OS and 143B cells, 3-HF can inhibit cell metastasis and reduce tumor growth in vivo [8,9]. However, its mechanism of action remains unclear. ZAK is a novel mixed lineage kinase-like protein containing a leucine-zipper (LZ) and a sterile-alpha motif (SAM) [10]. ZAK acts as a signal transduction kinase of the MAP3K family and encodes a protein with an N-terminal kinase catalytic domain, also termed MLTK (for MLK-like mitogen-activated protein triple kinase) [11,12]. Previous studies showed that there are two transcriptional splice variants encoding the different isoforms of ZAKα and ZAKβ, respectively; these isoforms have been characterized [11]. All of them have common structural characteristics that are unique among the protein kinase family: a catalytic domain bearing the amino acid motifs found in serine/threonine and tyrosine kinases, and one or two leucine-zipper motifs [13]. ZAKα contains a kinase domain followed by a short LZ motif and an SAM domain, and ZAKβ is composed of 455 amino acids and is identical to ZAKα from the N-terminus to the LZ motif; however, the ZAKβ sequence then diverges and lacks an SAM domain [11]. Numerous studies have indicated that ZAKα can play different roles in normal cells and in cancer cells. In our previous reports, we found that ZAKβ plays different roles in human OS cells and in H9c2 cardiomyoblast cells. In H9c2 cells, we found that ZAKβ plays an antagonistic role to antagonize and ameliorate the cardiac hypertrophic and apoptotic effects induced by ZAKα [13]. In addition, both the overexpression of ZAKβ in cardiac tissue and the beta-adrenergic stimulation of ZAKβ can activate the p38 and JNK pathways and lead to cardiac myocyte hypertrophy in transgenic mice [14]. ZAKβ can also activate JNK in response to saturated free fatty acids (FFA) [15]. In human OS cells, we found that ZAKβ could enhance ZAKα expression, resulting in a synergistic apoptotic effect [5,16]. In this study, we demonstrate that 3-HF can induce ZAK overexpression to trigger human OS cells apoptosis, and ZAKβ can play a leading role in activating ZAKα and a synergistic apoptotic effect.

## 2. Results

In the present study, we elucidated the effect of 3-HF against human OS cells through the ZAK*β* signaling axis.

### 2.1. 3-HF Reduces the Viability of Human Osteosarcoma Cells, which Correlates with Simultaneous Upregulation in ZAKα, β Levels and Cleaved Caspase Levels

**Figure 1:**
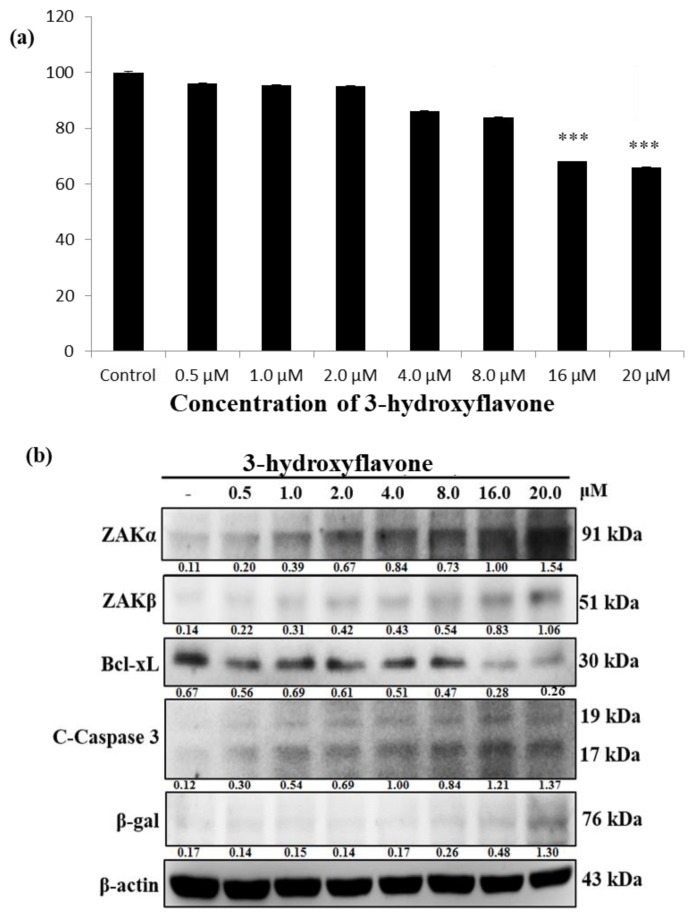
Dose-dependent effect of 3-Hydroxy-2-phenylchromone (3-HF) on human osteosarcoma (HOS) cells. (**a**) Effect of 3-HF treatment, which in a dose-dependent manner (0.5–20 μM) decreased the viability of human OS cells, measured with MTT assays. Data are shown as the mean ± SEM of three independent experiments. (**b**) Western blot analysis of the expression of Zipper sterile-alpha-motif kinase (ZAK), apoptotic protein C-Caspase 3, survival protein Bcl-xL and β-gal with 3-HF treatment in a dose-dependent manner (0.5–20μM). *** *p* < 0.001 represents significance with respect to the control group.

To investigate whether 3-HF inhibits cell viability of human OS cells, we performed an MTT assay, and the results show that the cell viability decreased as the concentration of 3-HF increased (Figure 1a). Subsequently, the Western blot analysis showed that ZAKα, β expression levels increased upon 3-HF treatment in a dose-dependent manner. In addition, the levels of cleaved-Caspase 3, a prominent apoptosis marker, increased with a simultaneous decrease in survival protein Bcl-xL. The senescence marker β-gal also increased with high concentrations of 3-HF (Figure 1b). According to the results, we confirmed the enhancing effect of 3-HF on ZAK expression and its influence in suppressing the viability of human OS cells.

### 2.2. 3-HF Can Trigger Cell Apoptosis and Decrease Mitochondrial Membrane Potential

**Figure 2:**
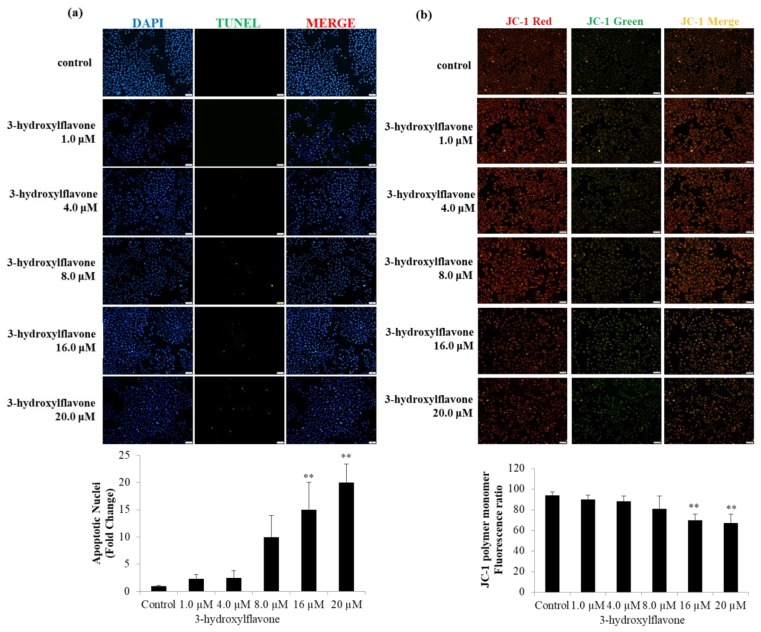

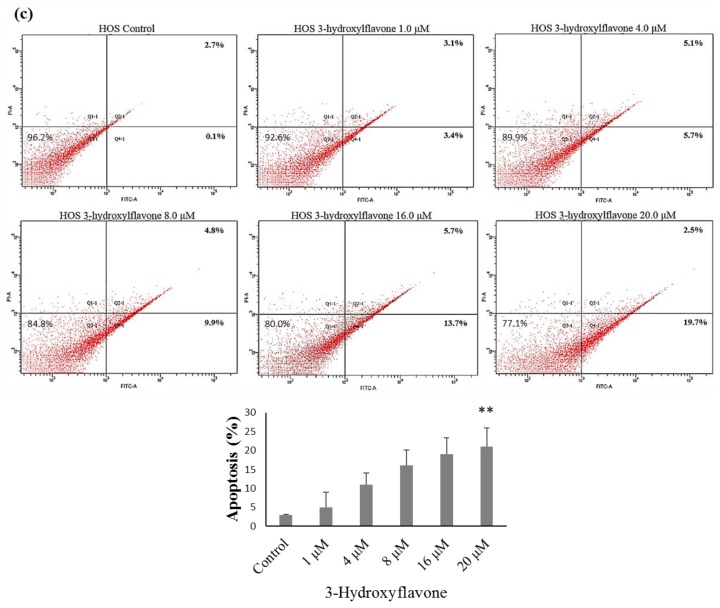
Effect of 3-HF on mitochondrial membrane potential and apoptosis in HOS cells. (**a**,**b**) Cells were seeded on 6-well plates and treated with 0, 1.0, 4.0, 8.0, 16.0, or 20.0 μM of 3-HF for 24 h. (**a**) The apoptotic effect detected by TUNEL assay and DAPI staining in human OS cells. (**b**) Fluorescence image of human OS cells stained with JC-1 after 24 h incubation with different concentrations of 3-hydroxyflavone. Photograph showing JC-1 red, JC-1 green and merge image. The JC-1 green fluorescence indicates a decrease in mitochondrial membrane potential, an event in apoptosis. Increased concentrations of 3-HF enhanced the loss of mitochondrial membrane potential. Scale bars indicate 100 µm at 20× magnification (**c**) The percentage of apoptotic cells in 3-HF groups increased significantly compared with the control group (parental cells). Flow charts: Q4, annexin V-positive and propidium iodide (PI)-negative cells indicate early apoptotic cells; Q2, annexin V- and PI-positive cells represent late apoptotic cells. ** *p* < 0.01 represents significance with respect to the control group.

Further, to understand the effect of 3-HF treatment on human OS cells, we used a TUNEL assay to detect apoptosis following 3-HF in human osteosarcoma cells. The results show that the apoptosis rate increased with 3-HF treatment in a dose-dependent manner compared with the control cells (Figure 2a). On the other hand, we used a JC-1 mitochondrial membrane potential assay to monitor mitochondrial health. These results showed a decrease in mitochondrial membrane potential through a reduction in red fluorescence, indicating an event of apoptosis under 3-HF treatment in a dose-dependent manner in human OS cells (Figure 2b). Simultaneously, we determined the proportion of apoptotic cells using a flow cytometer by the double staining of cultures with propidium iodide (PI) and annexin V-FITC. We found that dose-dependent increments in apoptotic cells among 3-HF-treated human OS cells were clearly demonstrated (Figure 2c). The proportion of apoptotic cells following 3-HF treatment increased significantly compared with the control group.

### 2.3. 3-HF Can Upregulate ZAK Expression to Induce Cell Apoptosis in Human OS Cells

**Figure 3:**
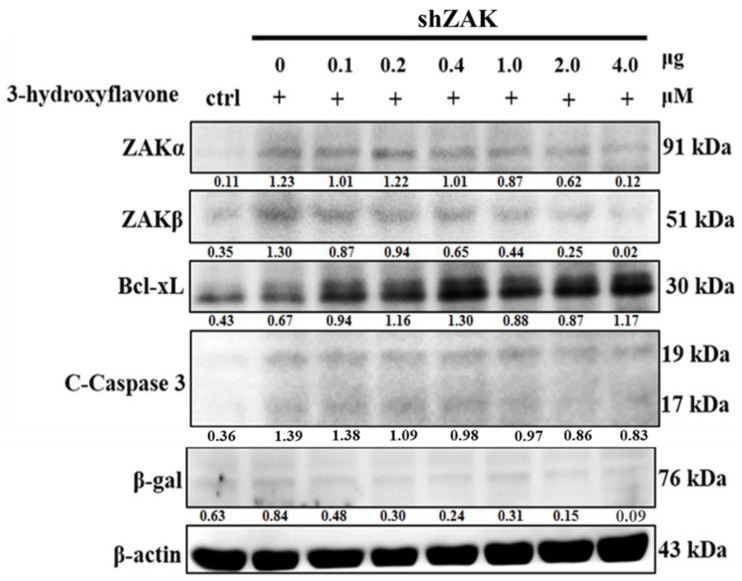
Role of ZAK in 3-HF-induced apoptosis in HOS cells. Western blot analysis of the expression of ZAK, apoptotic protein C-Caspase 3, survival protein Bcl-xL and β-gal by transient transfection of shZAK in a dose-dependent manner (0.1–4.0 μg/mL) following 3-HF treatment (20 μM).

According to the results in this study, 3-HF caused an increase in ZAK protein level, cell apoptosis and a reduction in cell survivability in human OS cells. To further confirm the effect of 3-HF in human OS cells, we transiently pre-transfected shZAK into human OS cells following 3-HF treatment. Based on the Western blot analysis, we found that the activation of ZAK and cell apoptosis induced by 3-HF were ameliorated by knockdown ZAK expression (Figure 3). Overall, the result shows that 3-HF can induce ZAK expression, resulting in an elevated apoptotic effect in human OS cells.

### 2.4. Cell Apoptosis Induced by 3-HF is Due to ZAKβ Overexpression not ZAKα in Human OS Cells

**Figure 4:**
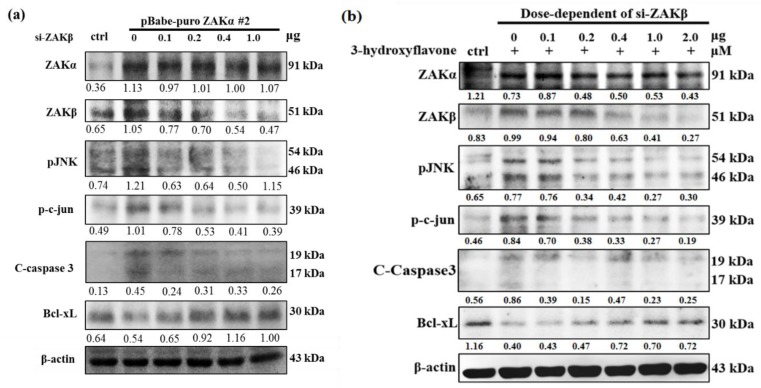
Role of ZAK transcriptional variants and the effect of 3-HF on ZAKβ and ZAKα. (**a**) Western blot analysis of the expression of ZAK, pJNK, p-c-Jun, apoptotic protein C-Caspase 3, and survival protein Bcl-xL with siZAKβ by transient transfection for 24 h in a dose-dependent manner (0–1.0 μg/mL) in a stable clone cell line expressing ZAKα. (**b**) Western blot analysis of the expression of ZAK, pJNK, p-c-Jun, apoptotic protein C-Caspase 3, and survival protein Bcl-xL with siZAKβ by transient transfection for 24 h in a dose-dependent manner (0–2.0 μg/mL) following 3-HF treatment in human OS cells.

In the previous studies, we demonstrated that ZAKβ might play a novel role in the regulation of ZAKα-dependent molecular mechanisms in cancer cells or normal cells. Here, we further hypothesize that ZAKβ plays a leading role to trigger cell apoptosis following 3-HF treatment in human OS cells. To confirm this hypothesis, we transiently transfected siZAKβ in stable clone cells with ZAK, which we set up in our previous research [5]. In the Western blot analysis, we found that knockdown ZAKβ eliminated the downstream signaling events mediated by ZAK in stable clone cells (Figure 4a). Subsequently, we transiently transfected siZAKβ following 3-HF treatment in human OS cells. Further, we found that knockdown ZAKβ can attenuate the downstream signaling events mediated by 3-HF in human OS cells (Figure 4b). Interestingly, we found that there was no significant effect on ZAKα protein expression (Figure 4a,b). Taken together, these results indicate that ZAKβ may play a major role in the regulation of molecular mechanisms in cancer cells or normal cells.

### 2.5. 3-HF Can Induce Cell Apoptosis by ZAKβ in Human OS Cells

**Figure 5:**
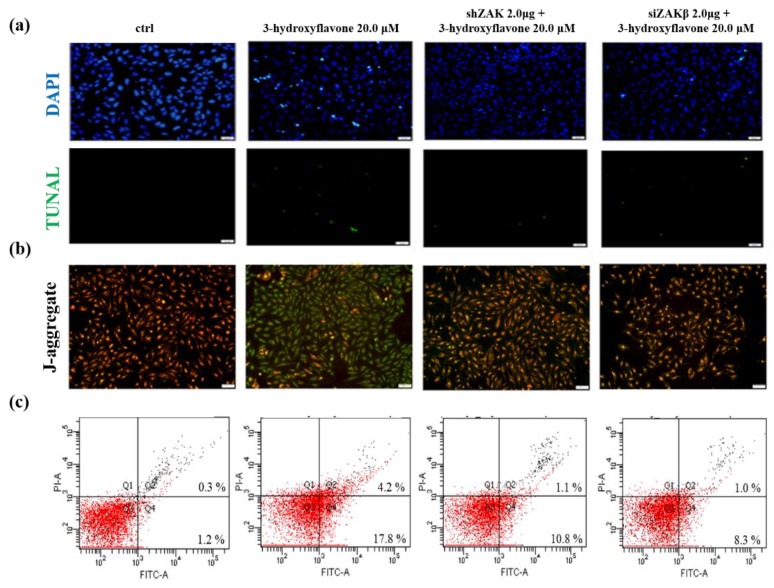
3-HF activates ZAKα to initiate a synergistic effect by both ZAKβ and ZAKα, resulting in aggravated apoptosis in HOS cells. (**a**,**b**) Cells were seeded on 6-well plates and pre-transfected with shZAK and siZAKβ for 24 h following 3-HF treatment in human OS cells. (**a**) The apoptotic effect was detected by TUNEL assay and DAPI staining in human OS cells. (**b**) Photograph showing JC-1 red, JC-1 green merged image. The JC-1 green fluorescence indicates a decrease in mitochondrial membrane potential, an event in apoptosis. Increased concentrations of 3-HF enhanced the loss of mitochondrial membrane potential. Transiently transfected shZAK and siZAKβ attenuated the loss of mitochondrial membrane potential following 3-HF treatment. Scale bars indicate 50 µm at 20× magnification. (**c**) The percentage of apoptotic cells in 3-HF groups increased significantly compared with the control group (parental cells) but decreased by transient transfection of shZAK and siZAKβ compared with the 3-HF treatment group. Flow charts: Q4, annexin V-positive and PI-negative cells indicate early apoptotic cells; Q2, annexin V- and PI-positive cells represent late apoptotic cells.

To further understand the mechanism of ZAKβ following 3-HF treatment in human OS cells, we used the TUNEL assay to detect apoptosis following 3-HF with pre-transient transfection of shZAK and siZAKβ in human osteosarcoma cells. Based on the fluorescence microscopy results, we determined that apoptosis increased with 3-HF treatment and decreased under ZAK knockdown, especially ZAKβ knockdown (Figure 5a). We also used a JC-1 mitochondrial membrane potential assay to monitor mitochondrial health. The fluorescence microscopy results showed a loss of mitochondrial membrane potential, indicating increased apoptosis under 3-HF treatment. However, the effects were attenuated in shZAK transfected cells, especially siZAKβ in human OS cells (Figure 5b). Moreover, the proportion of apoptotic cells following 3-HF treatment increased significantly, and the proportion of apoptotic cells following shZAK or siZAKβ transfection of cells under 3-HF treatment decreased significantly as compared with the control group (Figure 5c). Taken together, these results indicate that 3-HF treatment in human OS cells can induce cell apoptosis via the activation of ZAKβ.

## 3. Discussion

Osteosarcoma is an aggressive tumor that accounts for about 5% of pediatric malignancies. The majority of patients are curable with combined treatment of chemotherapy and surgery [17,18]. The standard chemotherapy treatment for osteosarcoma is based upon pre-operative and post-operative chemotherapy with doxorubicin, cisplatin, and methotrexate [19]. Osteosarcoma is generally considered as a highly invasive metastatic cancer with poor prognosis. The metastatic onset occurs during the early stages and in post-surgery, and therefore 85% of patients with osteosarcoma experience cancer metastasis [20,21,22]. Combinational treatment with surgery, chemotherapy, and radiation therapy is generally considered clinically. High-dose chemotherapy has shown improvements with overall three-year survival rates of over 79%, and combinational therapy improves it to over 85% [23]. However, the multifactorial drug resistance in osteosarcoma hampers the treatment efficiency and affects prognosis, therefore the long-term survival of osteosarcoma patients with recurrence and metastasis is still poor [24]. In this context, strategies to enhance the efficiencies of chemotherapy regimens with novel combinatorial therapy are desirable.

The previous studies on U2OS and 143B show that 3-HF inhibits tumor growth and metastasis in vivo [8]. In addition, 3-HF can inhibit endogenous Aurora B and induce growth inhibition of the cancer cell line [7]. However, the mechanism in human OS cells remains unclear. In our previous publication, we demonstrated that doxorubicin can induce cell apoptosis by overexpression of ZAK in human OS cells [5]. Furthermore, we have also demonstrated that ZAKβ can enhance ZAKα expression in human OS cells, resulting in a synergistic apoptotic effect [16]. However, due to the adverse side effects associated with doxorubicin, various alternative drugs are under consideration [25,26]. Moreover, about 40%–45% of patients with high-grade osteosarcoma are either only partially responsive or completely unresponsive to doxorubicin (Dox), due to the increased drug efflux by the transporter protein ABCB1 [19].

**Figure 6:**
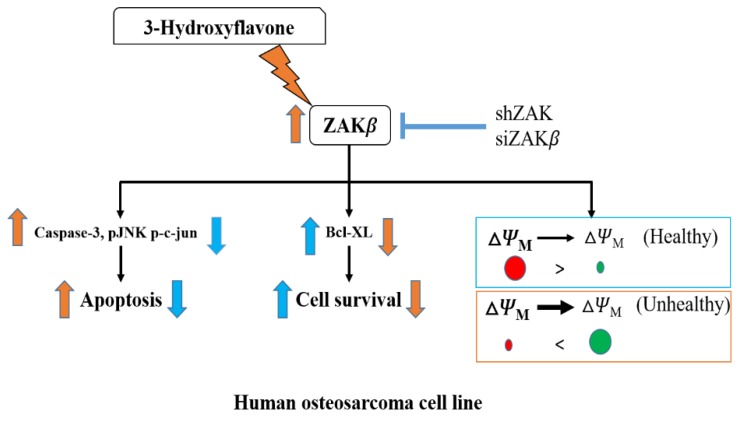
Schematic diagram showing the effect of 3-HF on human OS cells apoptosis. Arrow represents proteins modulated by 3-HF or shZAK and siZAKβ.

In this study, we provided evidence that 3-HF can induce cell apoptosis by upregulation of ZAKβ (Figure 6). In correlation with our previous report, 3-HF induced an increase in ZAKα, β levels, resulting in reduced cell viability and an increase in cell apoptosis in human OS cells. ZAK has been reported as a tumor suppressor protein in cancers of other origin [10,27]. Similar to our present results, overexpression of ZAK has been previously reported to elevate apoptosis in hepatoma cells [10]. Kinase activity of ZAK has also been demonstrated to trigger G2 arrest, thereby reducing proliferation in ZAK-expressing cells [13]. Therefore, ZAK is an ideal target to enhance the effect of chemotherapy against osteosarcoma, and since 3-HF selectively promotes ZAKβ expression, it causes a domino effect on apoptosis via ZAKα. Consequently, it is a potential candidate for further studies in this direction.

In conclusion, our results confirm the hypothesis that 3-HF can induce human OS cell apoptosis, mainly via ZAKβ, not ZAKα. According to this finding, ZAKβ may play a novel role in the clinical therapy for osteosarcoma.

## 4. Materials and Methods

### 4.1. Cell Culture

The human OS cell line was purchased from the American Type Culture Collection (ATCC, CRL-1543) (Rockville, MD, USA). Human OS cells were grown in Eagle’s Minimum Essential Medium (Sigma, St. Louis, MO, USA) containing 10% fetal bovine serum (Clontech, Mountain View, CA, USA) and 1% Pen-Strep Ampho (CORNING, Flintshire, UK) in humidified air (5% CO_2_) at 37 °C.

### 4.2. Whole Cell Extraction

Cultured human osteosarcoma cells were trypsinized and washed once with PBS. Then, the cell pellet was collected and lysed in lysis buffer (50 mM Tris (pH 7.5), 0.5 M NaCl, 1.0 mM EDTA (pH 7.5), 10% glycerol, 1 mM β-ME, 1% IGEPAL-630 and proteinase inhibitor) and centrifuged at 12,000 rpm for 30 min. Then, the supernatant was collected in a new 1.5 mL Eppendorf tube and stored at −20 °C.

### 4.3. MTT Assay

Cell viability was examined by the use of a 3-(4,5-dimethylthiazol-2-yl)-2,5-diphenyltetrazolium bromide assay. Human osteosarcoma cells (1 × 10^4^) were seeded on 24-well plates with MEM medium. The following day, the cells were treated with different concentrations of 3-HF and cultured for 24 h. Then, cell viability was assessed using the MTT reagent at a concentration of 5 mg/mL. Following incubation at 37°C for 3 h, the reaction was stopped by adding 200 μL of dimethyl sulfoxide (DMSO). After the crystals were dissolved, the absorbance of each sample was determined at O.D. 570 nm.

### 4.4. Transient Transfection

ShRNA-mediated ZAKα plasmid DNA was kindly provided by Dr. J. J. Yang (Chung Shan medical university, Taichung, Taiwan). The cells were grown to 60% confluence by the day of transfection. The shRNA-mediated ZAKα was transfected into human osteosarcoma cells using the PureFection transfection reagent according to the manufacturer’s guidelines (System Biosciences, Mountain View, CA, USA). After 24 h, the cells were harvested and extracted for analysis.

### 4.5. siRNA Transfection

Cells were plated in growth medium without antibiotics for 24 h prior to transfection. Transient transfection of siZAKβ was done using the PureFection transfection reagent according to the manufacturer’s instructions (System Biosciences, Mountain View, CA, USA). The cells were harvested 24 h after transfection.

### 4.6. Western Blot

The Western blot analysis was performed following previous reports [28]. Proteins were separated on a 10% SDS-PAGE gel and transferred to PVDF membranes. Non-specific protein binding was blocked in blocking buffer (5% milk, 20 mM Tris-HCl (pH 7.6), 150 mM NaCl, and 0.1% Tween-20), and proteins were blotted with specific antibodies in the blocking buffer at 4 °C overnight. After incubation with the secondary antibody for 1 h at room temperature, densitometric analysis of the immunoblots was performed using the AlphaImager 2000 digital imaging system (Digital Imaging System, Commerce, CA, USA). For repeated blotting, PVDF membranes were stripped with Restore Western Blot Stripping Buffer at room temperature for 10 min. The protein levels, represented by their band intensity, were determined by normalizing with the corresponding intensity of the internal control. The intensity was measured using imageJ 1.52v (NIH, Bethesda, MD, USA).

### 4.7. TUNEL

The APO-BrdU TUNEL Assay Kit (Roche, Basel, Switzerland) was used to perform the terminal transferase-mediated dUTP nick-end labeling of nuclei, following Wo et al. [19] and the manufacturer’s protocol.

### 4.8. JC-1 Staining

The mitochondrial membrane potential was assessed using the fluorescent probe JC-1 dye purchased from Sigma. Briefly, human OS cells were pre-transfected with shZAK and siZAKβ individually, and then treated with 3-HF and incubated with JC-1 working solution for 20 min at 37 °C in the dark. Cells were washed with cold JC-1 staining buffer, placed on ice, and observed immediately by fluorescence microscopy.

### 4.9. Detection of Cell Apoptosis Using Flow Cytometry

The detection of apoptosis in human OS cells, which were treated with 3-HF in a dose-dependent manner and transiently transfected with shZAK and siZAKβ following 3-HF treatment, was analyzed by determining the ratio of cells along with the nucleus concentration and fragment. Cells were collected after incubation and then suspended in the buffer. During the apoptosis assay, the cells were stained with propidium iodide and annexin V-FITC (BD Biosciences, San Jose, CA, USA) and determined by flow cytometry.

### 4.10. Antibodies and Reagents

The following antibodies were used in this study: anti-pJNK, anti-p-c-jun, anti-Bcl-xL, anti-β-gal, and anti-β-actin (Santa Cruz Biotechnology, Dallas, TX, USA). Anti-C-Caspase-3 was purchased from Cell Signalling Technology (Danvers, MA, USA). The ZAK monoclonal antibody (M02) was purchased from Abnova (Taipei, Taiwan). 3-HF was purchased from Sigma. siZAKβ was kindly provided by Dr. J. J. Yang (Chung Shan Medical University, Taichung, Taiwan).

### 4.11. Statistical Analysis

The data shown are provided as the means ± standard deviation (SD) of three (3) independent experiments. For comparisons between multiple groups, statistical analysis was performed by one-way ANOVA with Tukey’s post hoc test using GraphPad 5 statistical software (San Diego, CA, USA). *p* < 0.05 was considered as significant.

## Abbreviations

OS	Osteosarcoma
ZAK	Zipper sterile-alpha-motif kinase
∆Ψ_M_	Mitochondrial membrane potential
